# Mitochondrial energy metabolism is required for lifespan extension by the spastic paraplegia-associated protein spartin

**DOI:** 10.15698/mic2017.12.603

**Published:** 2017-11-30

**Authors:** Julia Ring, Patrick Rockenfeller, Claudia Abraham, Jelena Tadic, Michael Poglitsch, Katherina Schimmel, Julia Westermayer, Simon Schauer, Bettina Achleitner, Christa Schimpel, Barbara Moitzi, Gerald N. Rechberger, Stephan J. Sigrist, Didac Carmona-Gutierrez, Guido Kroemer, Sabrina Büttner, Tobias Eisenberg, Frank Madeo

**Affiliations:** 1 Institute of Molecular Biosciences, NAWI Graz, University of Graz, Graz, Austria.; 2 BioTechMed Graz, Graz, Austria.; 3 Kent Fungal Group, School of Biosciences, University of Kent, Canterbury, UK.; 4 Institute of Molecular and Translational Therapeutic Strategies (IMTTS), IFB-Tx, Hannover Medical School, Hannover, Germany.; 5 BioNanoNet Forschungsgesellschaft mbH, Graz, Austria.; 6 Omics Center Graz, BioTechMed-Graz, Graz, Austria.; 7 Institute for Biology, Freie Universität Berlin, Berlin, Germany.; 8 NeuroCure, Charité, Berlin, Germany.; 9 Equipe 11 Labellisée Ligue Contre le Cancer, Centre de Recherche des Cordeliers, Paris, France.; 10 Cell Biology and Metabolomics Platforms, Gustave Roussy Comprehensive Cancer Center, Villejuif, France.; 11 INSERM, U1138, Paris, France.; 12 Université Paris Descartes, Sorbonne Paris Cité, Paris, France.; 13 Université Pierre et Marie Curie, Paris, France.; 14 Pôle de Biologie, Hôpital Européen Georges Pompidou, Paris, France.; 15 Karolinska Institute, Department of Women's and Children's Health, Karolinska University Hospital Stockholm, Sweden.; 16 Department of Molecular Biosciences, The Wenner-Gren Institute, Stockholm University, Stockholm, Sweden.

**Keywords:** SPG20, mitochondria, metabolism, respiration, pyruvate dehydrogenase, cell death, aging

## Abstract

Hereditary spastic paraplegias, a group of neurodegenerative disorders, can be caused by loss-of-function mutations in the protein spartin. However, the physiological role of spartin remains largely elusive. Here we show that heterologous expression of human or *Drosophila* spartin extends chronological lifespan of yeast, reducing age-associated ROS production, apoptosis, and necrosis. We demonstrate that spartin localizes to the proximity of mitochondria and physically interacts with proteins related to mitochondrial and respiratory metabolism. Interestingly, Nde1, the mitochondrial external NADH dehydrogenase, and Pda1, the core enzyme of the pyruvate dehydrogenase complex, are required for spartin-mediated cytoprotection. Furthermore, spartin interacts with the glycolysis enhancer phospo-fructo-kinase-2,6 (Pfk26) and is sufficient to complement for *PFK26*-deficiency at least in early aging. We conclude that mitochondria-related energy metabolism is crucial for spartin’s vital function during aging and uncover a network of specific interactors required for this function.

## INTRODUCTION

Troyer Syndrome, a complicated form of hereditary spastic paraplegias (HSPs), is caused by mutations in the gene coding for spartin (*SPG20*), resulting in a dysfunctional protein. This loss-of-function disease is characterized by neurological and musculoskeletal symptoms 1. Spartin is conserved throughout multicellular eukaryotes, although expressed in all tissues, it seems to be of particular importance in neurons. This may explain the HSP-associated symptoms in the absence of the protein 2,3,4,5,6. The medical relevance of spartin goes beyond neurodegeneration, since the epigenetic silencing via hypermethylation of *SPG20* is associated with colorectal and gastric cancer. In fact, *SPG20* has been recently introduced as a new biomarker for these diseases 7,8.

Despite several proposed molecular functions of spartin, its precise role to regulate cellular processes remains poorly understood. Spartin carries at least four different protein-interacting and binding domains: the N-terminal microtubule-interacting and trafficking (MIT) domain, the C-terminal plant-related senescence domain (PSD), the ubiquitin binding region (UBR), and a protein-protein interaction PPxY motif 9,10,11. Diverse studies elucidating spartin’s cellular localization and interaction partners indicate a rather complex subcellular distribution 10,12,13,14,15,16,17,18,19,20. It is found in endosomes, the nucleus, the trans Golgi network, synaptogamin-positive vesicles, the spindle poles and the midbodies during mitosis, mitochondria, the ER, microtubules, and lipid droplets. It is also known to interact with ER- and mitochondria-associated heat shock proteins and proteins of the *endosomal sorting complex required for transport III* (ESCRT-III). Altogether, this suggests a plethora of diverse roles within the cell. Indeed, spartin has been postulated to impact a number of pathways. Spartin has been shown to impact synaptic growth and neuronal survival by regulating microtubule stability and endocytosis and by that to influence BMP (Bone Morphogenetic Protein)-signaling via its MIT domain 21,22,23,24. Furthermore, this MIT domain has been reported to interact with an ESCRT-III-associated protein Ist1, which recruits spartin to midbodies for subsequent successful abscission during cytokinesis 7,18,22. Several studies report a role for spartin in lipid droplet turnover, where it acts as an adaptor protein that activates and recruits ubiquitin ligases via its PPxY domain, thus promoting the ubiquitination of other lipid droplet-associated proteins 10,13,25. Additionally, it has been shown to be involved in the formation of aggresome-like structures by binding ubiquitin chains via the UBR domain 26. Interestingly, spartin binds to mitochondria via its PSD domain, but its function there remains elusive 15.

Yeast lacks an obvious DNA sequence-based *SPG20* homolog and therefore may offer a “clean room” to explore spartin’s functions. Importantly, yeast represents a well-established and powerful model organism to study metazoan proteins associated with neurodegenerative diseases 27,28,29,30,31,32,33. In addition, chronologically aging yeast cells display similar aging and cell death processes as aging post mitotic neuronal cells, and represent an intrinsic physiological model of eukaryotic cell death 34,35,36. Notably, mitochondria and their functions in energy metabolism and cell death are highly conserved from yeast to human 37,38,39. In this study, we made use of this and analyzed heterologous expression of *Drosophila* and human spartin in aging yeast, demonstrating a cytoprotective function of the protein that is connected to glycolytic and respiratory control.

## RESULTS 

### Spartin increases survival of aging yeast cells

**Figure 1:**
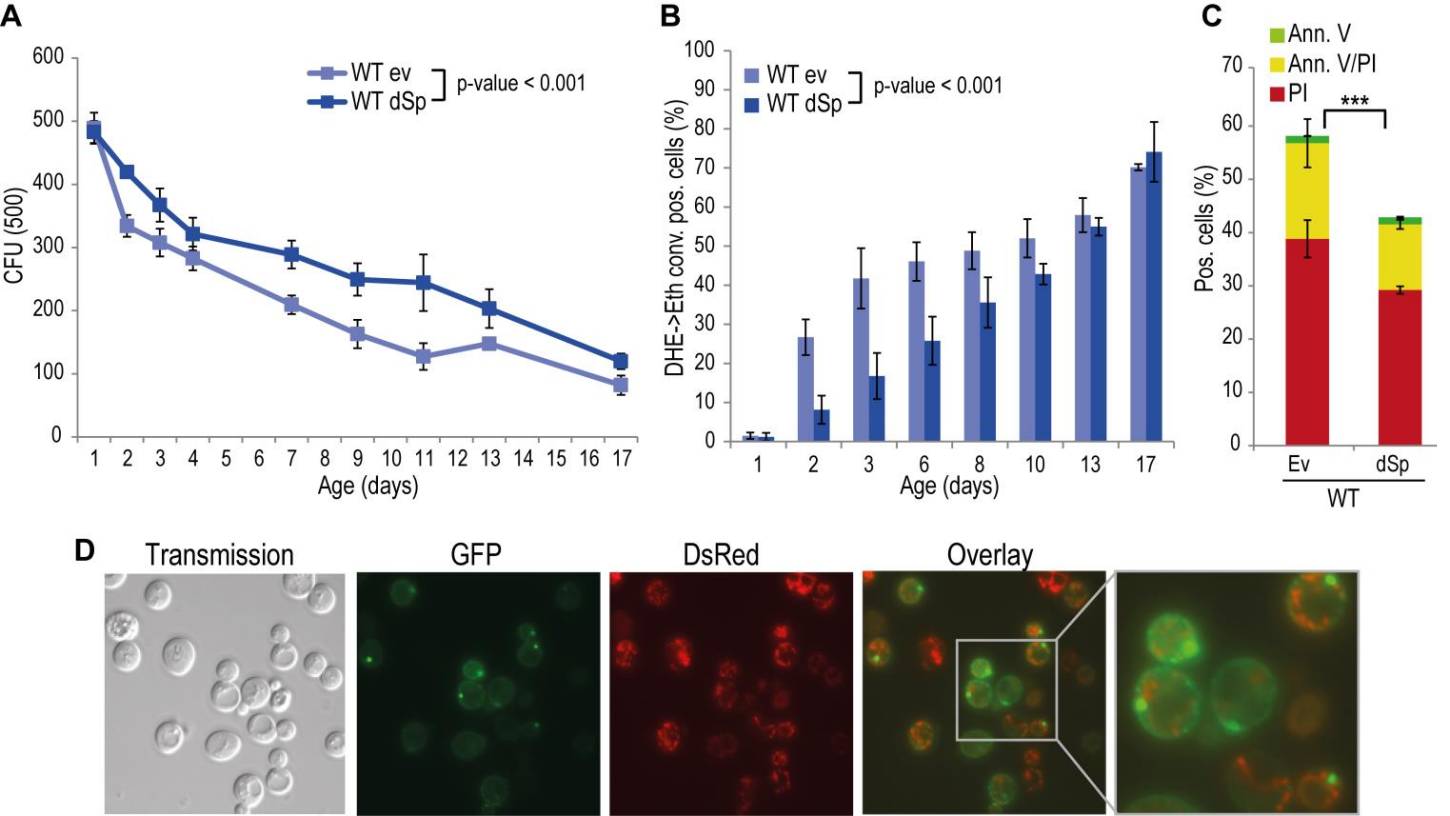
FIGURE 1: Spartin exerts a pro survival function in yeast. **(A)** Survival during chronological aging of WT cells expressing *Drosophila* spartin (dSp) or cells harboring the corresponding empty vector control (ev). Yeast cells were evaluated for clonogenicity at indicated time points after induction of expression by shift to 2% galactose containing media. Data represent mean values of at least four independent experiments performed at the same time. *CFU*, colony-forming unit.* Error bars* represent standard error of mean (SEM). **(B)** Quantification of oxidative stress levels (by conversion of dihydroethidium (DHE) to ethidium (Eth)). **(C)** phosphatidylserine externalization (apoptotic cell death) and loss of membrane integrity (necrotic cell death) using annexin V/propidium iodide (Ann.V/PI) costaining and flow cytometry-assisted quantification. **(D)** Fluorescence microscopy of WT cells expressing dSp^EGFP^ (green) and mitochondria visualized by co-expression with DsRed fused to a mitochondrial localization sequence on day 1 of chronological aging.

Spartin is present in organisms ranging from nematodes and flies to humans, and alignment of *Drosophila* and human spartin shows that the C-terminal PSD, the N-terminal MIT as well as the centrally located UBR domains are highly conserved (Fig. S2) 21. HSPs manifest in early childhood and progress during aging. Therefore we analyzed the impact of spartin on the aging process by heterologously expressing *Drosophila* spartin in yeast cells that were subjected to chronological aging. Expression of spartin in wild type cells (Fig. S1A) decreased age-associated clonogenic cell death (Fig. 1A) and reduced production of reactive oxygen species (ROS) (Fig. 1B), as quantified via superoxide-driven conversion of non-fluorescent dihydroethidium (DHE) into fluorescent ethidium 40,41. We next analyzed the mode of cell death affected by spartin expression and performed AnnexinV/PI co-staining, which allows to distinguish between early apoptosis (Annexin V+/ PI-), late apoptosis/secondary necrosis (Annexin V+/ PI+), and primary necrosis (Annexin V-/ PI+) 29,42. Spartin reduced markers of both apoptotic and necrotic cell death (Fig. 1C). Of note, while spartin exerted cytoprotection in every aging experiment performed, the kinetics of this cytoprotective effect varied slightly between the independent experiments.

### Spartin localizes in close proximity to mitochondria and interacts with proteins involved in energy metabolism

In many scenarios, cell death is strictly connected to mitochondria. Using a spartin-GFP fusion protein, we detected spartin in stationary cells either at the plasma membrane or in punctae in close proximity to mitochondria as visualized by co-expression with DsRed fused to a mitochondrial localization sequence (Fig. 1D). Co-immunoprecipitation followed by mass spectrometry corroborated spartin’s mitochondrial connection. Among the proteins that co-purified with spartin were several ones involved in vesicle transport (e.g. Yip3, an COPII vesicle interacting protein involved in ER to Golgi transport, the sorting nexin family member Atg20, which is required for cytosplama-to-vacuole targeting pathway and endosomal sorting as well as Sec4, a Rab family GTPase essential for vesicle-mediated exocytic secretion and autophagy) according to spartin’s proposed role in endosomal trafficking, but also a set of mitochondrial proteins (Table 1; Fig. S3A, B). Among those were regulators of mitochondria-associated proteostasis like Ssc1, an essential mitochondrial Hsp70 chaperone and component of the inner mitochondrial membrane translocase complex, and the cytosolic Hsp82/Hsc82, which are Hsp90 chaperones and among other functions important for outer mitochondrial membrane transport and the acetolactate synthase Ilv2. Further proteins involved in mitochondrial metabolism include the 6-phosphofructo-2-kinase Pfk26 and the mitochondrial external NADH dehydrogenase Nde1.

**Table 1:** Putative interaction partners of spartin revealed by mass spectrometry analysis of immunoprecipitated dSp-FLAG.

Sample	Protein	Dist. Peptides	kDa	Score	Location	Function
A	Ctr9	11	124.8	167.9	Nucleus	Paf1 complex - histon modification
	Pds5	2	147.0	19.8	Cytosol	Chromosome-associated
	Gde1	2	138.0	33.9	Cytosol	Glycerophosphocholine (GroPCho) phosphodiesterase
B	Pfk26	6	93.5	95.6	Cytosol	Regulator of glycolysis
	Rpn2	2	104.3	27.7	Cytosol	Proteasome
C	Spartin (fly)	4	63.0	58.4		
	Hsp/hsc82	2	81.4	31.8	Cytosol	Mitochondrial-associated chaperon
	Atg20	2	72.5	24.0	Cytosol	Vacuole-associated, cvt pathway
	Pfk26	11	93.5	182.0	Cytosol	Regulator of glycolysis
D	Spartin (fly)	4	63.0	290.7		
	Rtf1	3	65.9	48.0	Nucleus	Paf1 complex - histone modification
	Ssc1	5	70.8	76.6	Mitochondria	Chaperon
	Pfk26	3	93.5	43.1	Cytosol	Regulator of glycolysis
	Ilv2	2	74.9	29.9	Mitochondria	Isoleucine and valine biosynthesis
E	Spartin (fly)	17	63.0	54.8		
	Nde1	3	62.8	36.3	Mitochondria	NADH dehydrogenase
	Spartin (fly)	8	63.0	150.7		
	Cdc73	4	44.5	62.6	Nucleus	Paf1 complex - histone modification
	Tef2	12	50.0	185.7	Cytosol /nucleus	Translation
	Tef4	2	46.5	26.5	Cytosol /nucleus	Translation
	Rpl3	2	43.8	33.2	Cytosol /nucleus	Ribosome
	Bio3	5	53.7	84.5	Cytosol	Biotin biosynthesis
	Sam1	2	41.8	33.4	Cytosol	S-adenosylmethionine synthetase
	Rpt6	3	45.3	47.7	Cytosol	Proteasome
	Rpn5	3	51.8	37.5	Cytosol	Proteasome
	Rpn6	2	49.8	28.0	Cytosol	Proteasome
G	Sec4	2	23.5	34.6	Cytosol	Vesicle-mediated exocytic secretion and autophagy
H	Spartin (fly)	2	63.0	38.9		
	Rps18ap	8	17.0	121.8	Cytosol /nucleus	Ribosome
I	Rps14	6	14.7	92.3	Cytosol /nucleus	Ribosome
	Rpl26	6	14.2	85.0	Cytosol /nucleus	Ribosome
	Rpl23	4	14.5	71.8	Cytosol /nucleus	Ribosome
	Rps16	4	15.8	68.7	Cytosol /nucleus	Ribosome
	Rps13	4	17.0	58.6	Cytosol /nucleus	Ribosome
	Rps25	3	12.0	48.7	Cytosol /nucleus	Ribosome
	Rps19	3	15.9	44.3	Cytosol /nucleus	Ribosome
	Rpl27ap	3	15.5	42.1	Cytosol /nucleus	Ribosome
	Rpl14	2	15.2	35.8	Cytosol /nucleus	Ribosome
	Rps15	2	16.0	35.4	Cytosol /nucleus	Ribosome
	Rps12	2	15.5	33.6	Cytosol /nucleus	Ribosome
	Rpl33	2	12.2	27.0	Cytosol /nucleus	Ribosome
	Rps17	1	15.8	19.6	Cytosol /nucleus	Ribosome
	Yip3	4	19.4	68.3	Cytosol	COPII vesicles, involved in ER to Golgi transport

**Figure 2:**
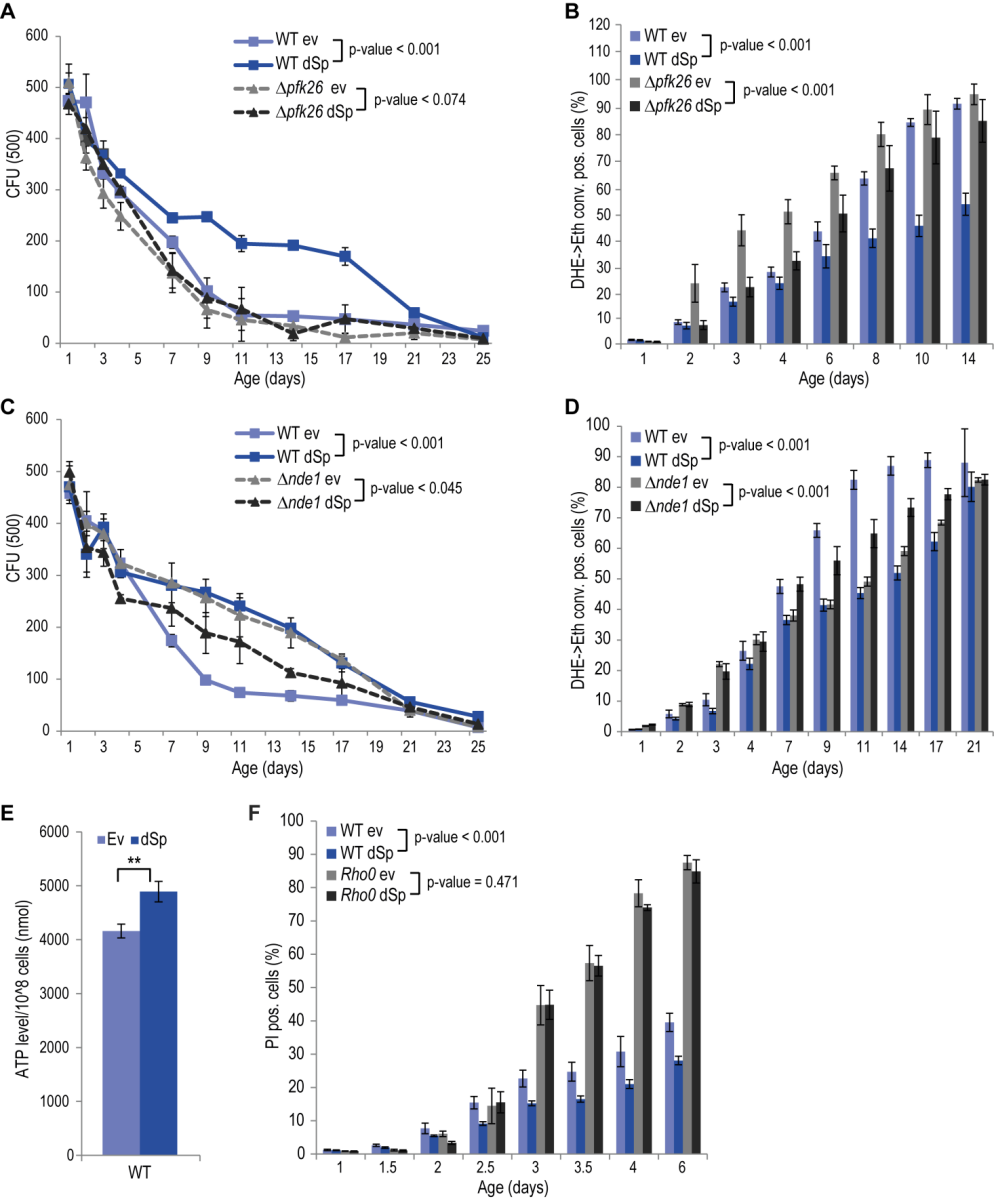
FIGURE 2: The pro-survival function of spartin depends on functional glycolysis and mitochondrial respiration. **(A, C)** Survival during chronological aging of WT, Δ*pfk26 and *Δ*nde1 *cells expressing dSp or cells harboring the corresponding ev. Data represent mean values of at least four independent experiments performed at the same time. *CFU*, colony-forming unit.* Error bars* represent standard error of mean (SEM). **(B, D)** Flow cytometry-assisted quantification of oxidative stress levels (conversion of DHE to Eth). **(E)** Quantification of ATP levels of WT cells expressing dSp or cells harboring the corresponding ev on day 2 of chronological aging. Data represent mean ± SEM of three independent experiments. **(F)** Cell death during chronological aging of WT and *rho^0^*cells expressing dSp or cells harboring the corresponding ev, quantified by measuring primary and secondary necrotic cells by PI staining, using flow cytometry. Data represent mean ± SEM of four independent experiments.

Given the central role of mitochondria in cellular energy metabolism and the fact that glycolytic metabolic networks are often dysregulated in neurodegenerative diseases, we decided to focus on two main energy metabolism-associated interaction partners, Pfk26 and Nde1. Pfk26 stimulates glycolysis via activation of Pfk1 that converts fructose-6-phosphate to fructose-1,6-bisphosphate, thus driving glycolysis to produce energy and the glycolytic end product pyruvate. We analyzed spartin expression upon deletion of *PFK26*, which per se caused an increase in ROS and cell death early during aging (Fig. 2 A, B). This could be prevented by spartin expression, however, only at the beginning, but not as aging progressed (Fig. 2 A, B). Deletion of *PFK27*, which codes for a Pfk26 paralogue specifically active during fermentation, had no effect on spartin-mediated cytoprotection (Fig. S4A, B). Therefore, Pfk26 seems to be specifically involved in the spartin effects. Expression levels of dSp were similar in all strains (Fig. S1B, S4B).

Another identified spartin interactor that is involved in metabolism was Nde1, which is the yeast complex I-like enzyme that provides electrons from cytosolic NADH to the mitochondrial respiratory chain. Interestingly, spartin expressed in cells lacking Nde1 lost its beneficial effects and even caused toxicity at later stages of aging (Fig. 2C, D). Quantification of total cellular ATP levels suggested increased ATP production upon spartin expression, pointing to enhanced respiratory capacity (Fig. 2E). To test whether mitochondrial function and, in particular, active respiration are a general requirement for the cytoprotective effects of spartin, we analyzed its effect in *rho^0^* cells, which lack mitochondrial DNA and are thus respiratory-deficient. Indeed, spartin-mediated cytoprotection was lost in the *rho^0^* background (Fig. 2F). Of note, expression levels of dSp were similar in all strains (Fig. S1B).

Altogether, spartin cytoprotection depends on the mitochondrial-glycolytic axis that determines cellular energy metabolism.

### The pyruvate dehydrogenase complex contributes to spartin-mediated protection

**Figure 3:**
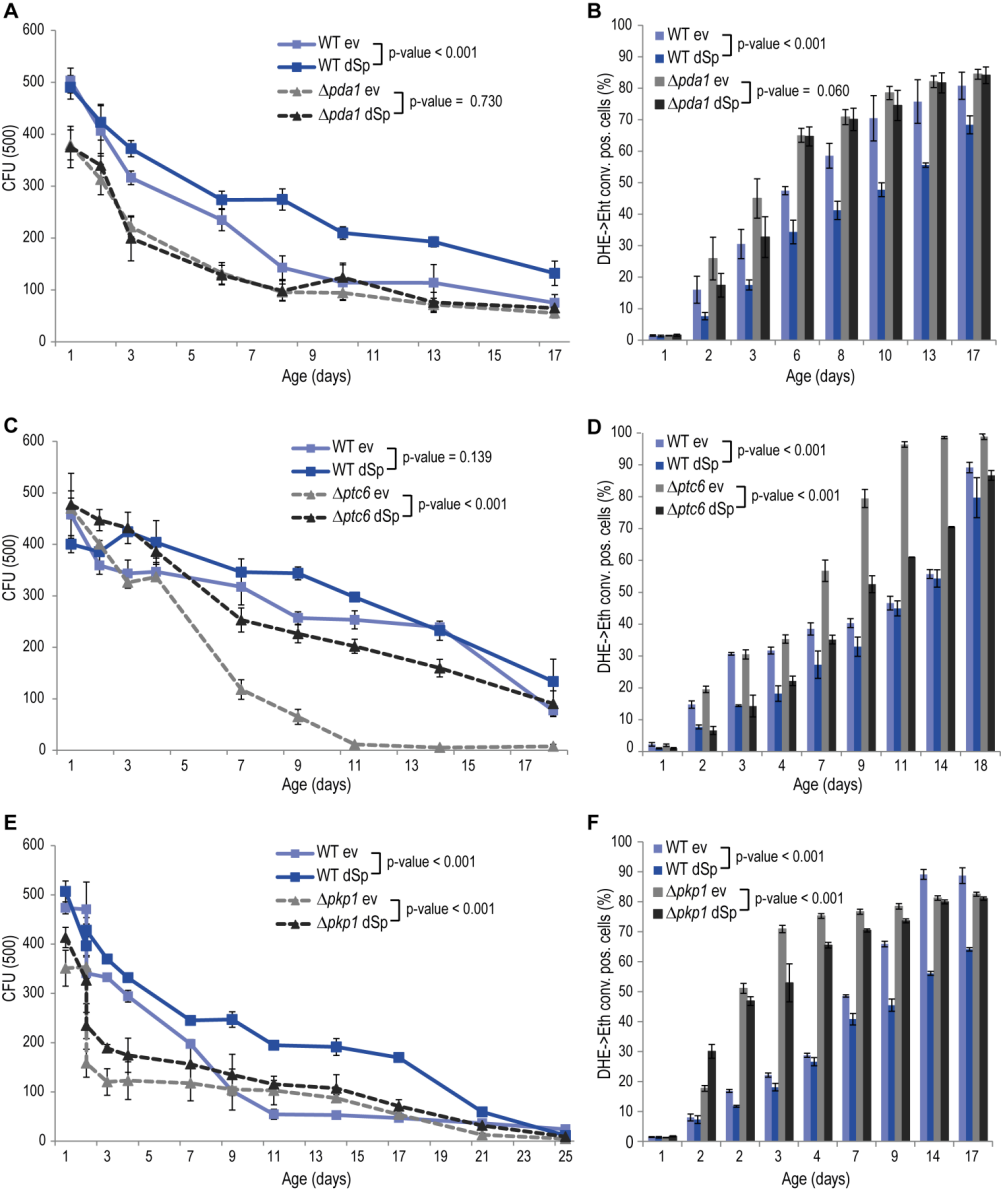
FIGURE 3: Spartin requires pyruvate dehydrogenase (PDH) complex activity for cell death protection in aging. **(A, C, E)** Survival during chronological aging of WT, D*pda1,* D*ptc6 or *D*pkp1 *cells expressing dSp or cells harboring the corresponding ev. Data represent mean ± SEM of four independent experiments. *CFU*, colony-forming units. **(B, D, F)** Quantification of oxidative stress levels (conversion of DHE to Eth) quantified by flow cytometry.

The glycolytic products NADH and pyruvate are imported from the cytosol into mitochondria to feed into oxidative phosphorylation. While mitochondrial NADH is converted by Nde1, pyruvate is transported into mitochondria by the pyruvate dehydrogenase (PDH) complex. Given the de-pendency of spartin on Nde1 (Fig. 2C, D), we next tested spartin expression in cells lacking Pda1, a core enzyme of the PDH complex essential for its function. Deletion of *PDA1* led to increased cell death and ROS production during chronological aging and to a complete abrogation of the cytoprotective capacity of spartin (Fig. 3A, B). The activity of the PDH complex is regulated by the phosphatase Ptc6 and the kinase Pkp1, which act on Pda1 and promote or inhibit PDH activity, respectively 14. We thus testedspartin expression upon deletion of these regulators. In *PTC6*-deficient cells, i.e. with decreased PDH complex activity, spartin expression was able to exert its beneficial effects (Fig. 3C, D). Notably, human spartin completely inhibited age-associated cell death upon *PTC6* deletion, suggesting a conserved role of this pathway (Fig. S5A, B). In the absence of the kinase Pkp1, i.e. under Pda1 inhibitory conditions, spartin expression first enhanced ROS production at early time points, but then displayed its usual rescuing effect later upon aging (Fig. 3E, F). In summary, these data suggest that spartin can promote PDH complex activity as long as the Pda1 core enzyme remains functional.

Besides its function as an activator of the PDH complex, Ptc6 is known to activate mitophagy via induction of the retrograde response 43. Mitophagy is a mitochondria-specialized form of macroautophagy, an intracellular degradative process with mostly cytoprotective properties 44. Thus, we tested for a potential connection between this additional role and spartin-mediated cytoprotection. Quantification of the retrograde response by a *CIT2* promotor activity assay demonstrated that spartin has no impact on the retrograde response in aging wildtype or (*ptc6 *cells (Fig. S6A, B). Also the deletion of *PTC6* per se did not affect the retrograde response compared to wildtype in our experimental aging conditions (Fig. S6A, B). Of note, these results differ from studies showing an active role of Ptc6 in mitophagy/retrograde response 43,45. This discrepancy might be due to differing experimental setups: we used galactose as the carbon source while previous studies used lactate or caffeine, both known to induce mitophagy. Thus, in cells aging on galactose, the lack of Ptc6 causes premature death that is independent of the role of this phosphatase in retrograde response, and expression of spartin can complement for the loss of this vital Ptc6 function(s). Of note, expression levels of dSp were similar in all strains (Fig. S1C).

## DISCUSSION

Mutations in *SPG20* lead to loss of function of the protein spartin, which results in Troyer Syndrome. In this study we investigated the role of heterologously expressed *Drosophila* and human spartin during chronological aging of yeast cells, a model of post-mitotic cellular aging in eukaryotes. Using clonogenicity assays combined with the examination of cell stress markers, we demonstrate that spartin has a pro-survival function. Spartin reduced ROS production and increased lifespan by interacting with and regulating key players of mitochondria-associated metabolism. Previous studies on spartin mainly focused on the characterization of the protein’s domains and motifs, interactors and localization as well as on the pathological consequences of its mutations. However, the vital functions of spartin yet remain poorly understood. In this work, we suggest that spartin has important vital functions by improving mitochondria-associated metabolism.

Several lines of evidence suggest that spartin might be functionally associated with mitochondria, a central organelle for metabolism and cell death control. In fact, spartin has been shown to translocate to mitochondria 15,16,17,46. Cells with decreased spartin levels display abnormal mitochondrial morphology and mobility as well as a reduction of mitochondrial membrane potential and calcium uptake 15,46. Recently, muscle biopsy of patients with mutated *SPG20* revealed reduced cytochrome c oxidase (COX) activity leading to decreased oxidative phosphorylation 6. Interestingly, another HSP-associated gene, *SPG7*, which codes for the metalloproteinase paraplegin, is part of a complex in the inner mitochondrial membrane. Absence of *SPG7* leads to reduced complex I activity and decreased stress tolerance in humans 47. Further, muscle biopsies of HSP patients with mutated *SPG7* show defects in mitochondrial oxidative phosphorylation 47. Still, the possibility and nature of a putative mitochondrial function of spartin remains unclear.

Using fluorescence microscopy as well as pulldown analysis, we have discovered that a certain amount of spartin localizes to the proximity of mitochondria and interacts with mitochondrial proteins in yeast. For instance, we identified the mitochondrial external NADH dehydrogenase, Nde1, as a potential interactor of spartin. Nde1 is part of a supercomplex that replaces complex I in yeast and provides cytosolic NADH to the mitochondrial respiratory chain 48. In (*nde1* cells, spartin failed to display its pro-survival function, suggesting that spartin’s interaction with Nde1 is crucial for its beneficial effects on cell survival. In addition, we found the cytosolic glycolytic flux-enhancing phospho-fructo-kinase Pfk26 to interact with spartin. Our results further show that spartin’s vital functions partly depend on this interactor. Interestingly, spartin can prevent ROS accumulation in the absence of Pfk26 only during early aging. Thus, different pathways might be influenced at different stages of the aging process, which remains to be elucidated. Finally, we also found that mitochondria-associated chaperones (i.e. Ssc1 and Hsp82/Hsc82) co-purify with spartin. This is in line with published data, showing that spartin interacts with a human mitochondrial Hsp70 (Grp75) 17. Interestingly, the Grp75 yeast homolog (Ssq1) cooperates with yeast Ssc1 49. Given that mitochondrial chaperones are important for protein import and maturation of certain mitochondrial complexes, these chaperones might be directly linked to the mitochondria-associated vital functions of spartin. Such dependency needs to be mechanistically elucidated in future work.

Given the impact of spartin on mitochondria, we tested spartin’s effects in the absence of Pda1, a core enzyme of the PDH complex, which in turn is necessary for mitochondrial import of pyruvate to feed into oxidative phosphorylation and thus links glycolysis to mitochondrial energy metabolism. The pro-survival effect of spartin was lost in Pda1-deficient cells. Interestingly, the rescuing effect became even more pronounced in cells lacking Ptc6, an activator of the PDH complex. Also the absence of the PDH-inhibitory kinase Pkp1 did not abolish the rescuing effects of spartin. Thus, the effects of spartin rather rely on the core catalytic subunit of the PDH complex (Pda1) than on its known regulators. Whether the actual PDH activity is modulated by spartin directly or via indirect alternative routes remains to be analyzed.

**Figure 4:**
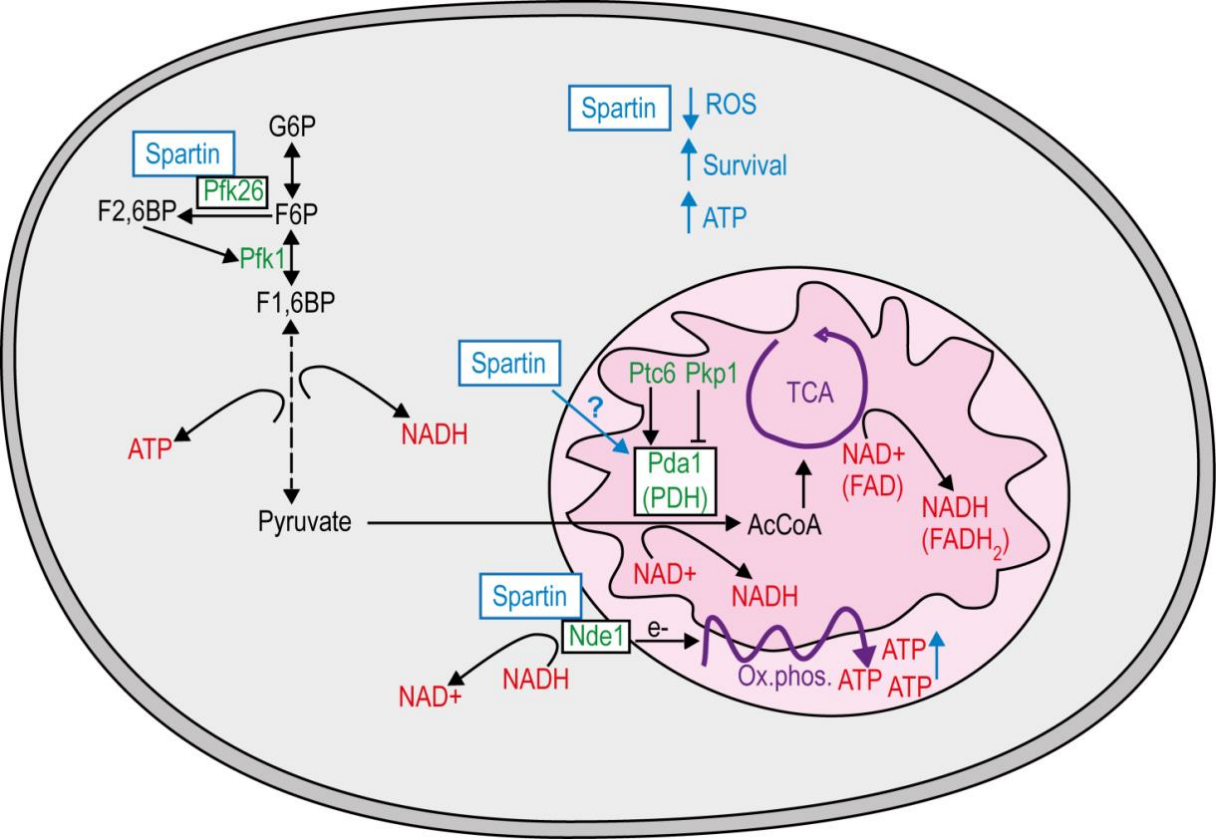
FIGURE 4: Model of how spartin modulates mitochondrial energy metabolism. Spartin has a pro-survival effect in chronological aging yeast and requires key regulators in energy metabolism. Spartin interacts with the cytosolic glycolytic flux-enhancing phospho-fructo-kinase Pfk26 and partly depends on Pfk26 to promote longevity, possibly by regulating the availability of NADH and pyruvate for also mitochondrial metabolism. The mitochondrial external NADH dehydrogenase Nde1 oxidizes NADH to transfer electrons to complex II and thus promotes oxidative phosphorylation to produce ATP. Spartin interacts with Nde1 and fails to exert its pro-survival role in (*nde1* cells. The pyruvate dehydrogenase (PDH) complex converts pyruvate to acetyl-CoA (AcCoA), which feeds the tricarboxylic acid (TCA) cycle to produce NADH and FADH_2_ serving again ATP production through oxidative phosphorylation. Pda1, the core enzyme of the PDH complex can be activated by phosphorylation through the phosphatase Ptc6 and inactivated by Pkp1 kinase. In the absence of Pda1 spartin does not promote longevity, but it can completely complement cell death due deletion of *PTC6 *and therefore mimics Ptc6 function. Whether spartin directly or indirectly (through associated metabolism) modulates PDH to promote cell survival remains to be investigates, In the presence of spartin cells may thus produce more ATP due to optimized oxidative phosphorylation. *G6P*, glucose-6-phophate. *F2,6BP*, fructose-2,6-biphosphate. *F6P*, fructose-6-phosphate. *Pfk1*, Phosphofructokinase 1. *F1,6BP*, fructose-1,6-biphophate.

Nde1 as well as the PDH complex link cytosolic glycolysis to mitochondria. In fact, we show here that spartin increases ATP production and depends on intact mitochondria to exert its vital functions (Fig. 4). This goes in line with the current literature. For instance, enhanced respiration has been demonstrated to act cytoprotectively in neurodegenerative settings 50. Nde1 and the PDH complex might function as a metabolic regulation handle controlled by spartin. Highly active Nde1 and PDH decrease both NADH and pyruvate levels in the cytosol, which simultan-eously increase respiration and glycolysis 51. Additionally, Pfk26 is the known positive regulator of glycolytic flux and might be influenced by its interaction with spartin. Thus, one of the primary functions of spartin could reside in the regulation of glycolytic to mitochondrial respiration-associated metabolic flux via interaction at several check points.

Besides its basic function in the mitochondrial respiratory chain, NAD^+^/NADH metabolism is also part of metabolic adaptations known to influence aging, cell death and oxidative stress. For instance, the NAD^+^/NADH equilibrium between cytosol and mitochondria determines the longevity effect in response to caloric restriction (CR) 52. Notably, CR-induced longevity also depends on actively respiring mitochondria 53. Moreover, an imbalance of the cytosolic NAD^+^/NADH ratio due to the dysregulation of the glycolytic metabolic network has been linked to several diseases, including cancer 54. Intriguingly, another study proposed spartin as a biomarker in certain types of cancer, in which hypermethylation of *SPG20* and thus downregulation of spartin expression correlates with tumorigenesis 7,8. This might be associated with the Warburg effect, by which cancer cells shift their metabolism from mitochondrial respiration to aerobic glycolysis 55. Finally, neurons highly rely on mitochondrial respiration to serve their energy needs, which is accompanied by low levels of glycolysis. Interestingly, spartin seems to be able to control all key regulators in this metabolic adaption processes. Pfk26 is the key regulator of glycolytic flux, whereas Nde1 and PDH are the bottlenecks to serve mitochondrial respiration.

Our study supports the assumption and adds evidence to the hypothesis that mitochondrial dysfunction is crucial in the pathogenesis of Troyer Syndrome. Moreover, it shows that spartin is an important key regulator of longevity-associated metabolic status of the cell, which needs further investigation in future research.

## MATERIALS AND METHODS

### Yeast strains, plasmids and media

Experiments were carried out in BY4741 wild type (MATa *his3Δ1 leu2Δ0 met15Δ0 ura3Δ0) *and corresponding null mutants from the EUROSCARF strain collection Δ*nde1, *Δ*pfk26, *Δ*pda1, *Δ*pkp1, *Δ*ptc6 and *Δ*rtg2, as well as in rho^0 ^cells, lacking mitochondrial DNA *29. Gene expression was under the control of galactose-regulated promoters and therefore strains were precultured on SC medium containing 0.17% yeast nitrogen base (Difco, BD Biosciences, Schwechat, Austria), 0.5% (NH_4_)_2_SO_4_ and 30 mg/l of all amino acids (except 80 mg/l histidine and 200 mg/l leucine), 30 mg/l adenine and 320 mg/l uracil with 2% glucose (SCD) and respectably shifted to 2% galactose (SCG). All amino acids were purchased from Serva (research grade, ≥98.5%). Transformation was done using the lithium acetate method 56. Notably, at least three different clones were tested in each experiment, to rule out clonogenic variation. For heterologous expression of *Drosophila* or human spartin, genes were amplified by PCR from respective cDNA and inserted into pESC-HIS vector from Stratagene (galactose promotor and c-terminal FLAG-tag) or pUG35-URA vector (methionine-repressible promoter and c-terminal GFP-tag) for microscopy. For *Drosophila* spartin (dSp) primers 3’-ATC TAC TAG T AT GGC GGA GGA GGA AAG C-5’ and 3’-ATC TAT CGA TTT CCT TCA AAG CTC GAA GAT CCG-5’ (pUG35-URA) or 3’-ATC T AT CGA TGT TTC CTT CAA AGC TCG AAG ATC CG-5’ (pESC-HIS), for human spartin (hSp) primers 3’-ATC TGA ATT CAT GGA GCA AGA GCC ACA AAA TG-5’ and 3’-ATC TGA ATT CTT TAT CTT TCT TCT TTG CCT CCT TTA CTT CC-5’ (pUG35-URA) or 3’-ATC TGC GGC CGC TTT ATC TTT CTT CTT TGC CTC CTT TAC TTC C-5’ (pESC-HIS) were used. Resulting plasmids were verified by sequencing by eurofins/MWG and expression was verified by immunoblot analysis. For mitochondrial co-localization studies cells additionally harbored plasmid, expressing dsRed with a mitochondrial localization sequence constitutively (pFM123-LEU mito dsRed).

### *S. cerevisiae* immunoblot analysis

To verify proper and equal expression of comparing strains we harvesting an aliquot (3.5 OD) of cells cultured for the chronological aging experiments, 20 hours after shift to expression media (SCG). Preparation of cell extracts and immunoblotting were performed as described 42. Soluble proteins were separated on Tricine-SDS polyacrylamide gels with an acrylamide concentration of 12,5%, transferred on Polyvinylidendifluorid (PVDF) membranes, and probed with murine monoclonal antibodies against FLAG-epitope (Sigma), murine monoclonal antibodies against glyceraldehyde-3-phosphate dehydrogenase (kind gift from Günther Daum) and the respective peroxidase-conjugated affinity-purified secondary antibody (Anti-Mouse IgG-Peroxidase antibody A9044, Sigma). For detection the ECL system was used (Amersham). Immunodetection was done using luminol. Membranes were digitized in ChemiDoc^TM^ Touch Imaging System (BIO RAD).

### Chronological aging, clonogenictiy, and test for stress and cell death markers

For chronological aging experiments cells were inoculated in SCD media to an OD_600_ of 0.05 and cultured at 28°C and shaking at 145 rpm. Cells containing the pESC-HIS vector were after grown for 4.5 hours to an OD_600_ of 0.15-0.2 then shifted to expression media containing galactose (SCG). At indicated time points cell survival was determined by clonogenictiy. Therefore cell cultures were counted with a CASY cell counter (Schärfe System) and 500 cells were plated on YPD agar plates (2% peptone, 1% yeast extract, 2% glucose, and 2% agar) and incubated for 2 to 3 days at 28°C. Subsequently colony forming units were counted using an automated colony counter. Additionally, oxidative stress was determined by measuring the conversion of dihydroethidium (DHE, Sigma-Aldrich) to the red fluorescent ethidium and subsequent flow cytometry analysis (BD FACSAria). Cell death was measured via propidium iodide (PI, Sigma-Aldrich) staining in cells that have lost their plasma membrane integrity and quantified with flow cytometry analysis. Further, Annexin V/PI co-staining (Annexin V-FLUOS Staining Kit, Roche Applied Science) was used at indicated time points and quantified with flow cytometry analysis to distinguish between early apoptotic (Annexin V positive), late apoptotic (secondary necrotic, double positive), as well as necrotic cells (only PI positive) cells. 30,000 cells per sample were evaluated using BD FACSDiva software. Representative aging analyses are shown with at least three independent cultures aged at the same time. All aging analyses were performed at least twice in total with similar outcome.

### Microscopy

Microscopy of GFP expressing cells was performed with a *Zeiss Axioskop* microscope using a *Zeiss Plan-Neofluar* objective lens with 63× magnification and 1.25 numerical apertures in oil (using Zeiss Immersol) at room temperature. Fluorescence microscopic sample images were taken with a *Diagnostic Instruments* camera (Model: SPOT 9.0 Monochrome-6), acquired and processed (coloring) using the Metamorph software (version 6.2r4, Universal Imaging Corp.) Mitochondria were visualized by co expression of mitochondrial-targeted dsRed (pFM123-LEU).

### Quantification of ATP levels

To determine the ATP level of yeast cultures, intracellular metabolites were obtained using hot ethanol extraction. ATP was measured using the ATP Determination Kit (Molecular Probes, Life Technologies). This assay is based on an ATP-dependent reaction of recombinant firefly luciferase, which induces bioluminescence of its substrate D-luciferin and is directly correlated with the ATP content. The luminescence was measured at a GloMax. Data analysis was performed with Microsoft Excel and Graphpad Prism. All data were normalized to the number of living cells within the samples.

### Quantification of retrograde response

Retrograde response (RTG) was monitored by *CIT2* promotor activity 57. Strains were transformed with and selected for plasmid pCG479 containing lacZ under the control of the *CIT2*-promotor (kind gift from Gourlay Campbell). Briefly 1×10^7^ cells were harvested and resuspended in Z-buffer (0.06 M Na_2_HPO_4_*7H_2_O, 0.04 M Na_2_H_2_PO_4_*2H_2_O, 0.01 M KCl, 0.0001 MgSO_4_, pH 7.0) and 2.5 mM beta-mercaptoethanol. With an aliqout of this suspension OD_600_ was measured on the GENiosPro 96-well fluorescence plate reader (Tecan, Grödig, Austria). Cells for the assay were vortext for 30 seconds containing additional SDS with final concentration of 0.01% and 10% CHCl_3_. For the reaction 220 µg ONPG (in Z-buffer solution) was added as substrate and incubated until solution got yellowish. Time was noted and the reaction was stopped with 250 mM Na_2_CO_3_. An aliqout of the water-phase was measured on the Tecan at OD_405_. For calculating RTG activity RFU of OD_405_ was normalized on incubation time and OD_600_.

### Pulldown assay and LC-MS/MS analysis

Whole-cell extracts of cells expressing FLAG-tagged *Drosophila* spartin or cells harboring the corresponding vector control were lysed and subjected to pulldown analysis using agarose beads coupled to monoclonal anti-FLAG antibody (Anti-FlagTm M2 affinity gel, Sigma). Subsequent eluates were separated by SDS-PAGE using Tricine-SDS polyacrylamide gels. The gel lanes were cut into slices, which were in-gel digested with trypsin (Promega), and the resulting peptide mixtures were analyzed by LC-MS/MS. For detailed information see 58.

## SUPPLEMENTAL MATERIAL

Click here for supplemental data file.

All supplemental data for this article are also available online at  http://microbialcell.com/researcharticles/mitochondrial-energy-metabolism-is-required-for-lifespan-extension-by-the-spastic-paraplegia-associated-protein-spartin/.
