# Supplementary material for: Mitochondrial energy metabolism is required for lifespan extension by the spastic paraplegia-associated protein spartin
Source: Microb Cell. 2017 Nov 30;4(12):411–22. doi: 10.15698/mic2017.12.603 (PMC5722644; doi:10.15698/mic2017.12.603)
Supplement: Supplementary file 1 [file mic-04-411-s01.pdf]

Supplementary Figures

Supplementary Figure 1

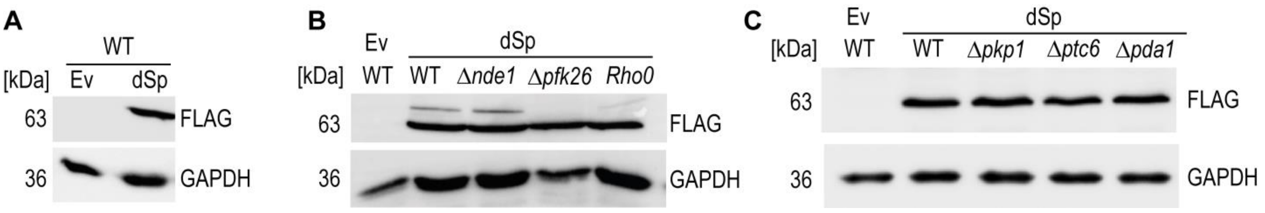

Supplementary Figure 1. Spartin expression is similar in all strains. *A.* Western blot analysis of dSp expression in WT and ∆*ptc6* cells, *B*. WT, ∆*nde1,* ∆*pfk26* and *rho0* cells and *C*. WT, ∆*pkp1,* ∆*ptc6* or ∆*pda1* cells. The blots were probed with FLAG and as a loading control with glyceraldehyde-3-phosphate dehydrogenase (*GAPDH*) antibodies and the corresponding secondary antibodies.

Supplementary Figure 2

```
Homo sapiens       MEQEPQNGEPAEIKIIREAYKKAFLFVNKGLNTDELGQKEEAKNYYKQGIGHLLR--GIS
D. melanogaster    MAEEE-SEFLEAYAGIRTAYKAAMTQVDLAVSHEEQESPGQAIVAYELALRMIEDTFGIP

Homo sapiens       IS-SKESEHTGPGWESARQMQQKMKETLQNVRTRLEILEKGLATSLQNDLQEVPKLYPEF
D. melanogaster    VGLPNKIDTVQAEWNDACALIQKLKSAETELRYRLKVLRSQKQSIDDSAVEATEE-----

Homo sapiens       PPKDMCEKLPEPQSFSSAPQHAEVNGNTSTPSAGAVAAPASLSLPSQSCPAEAPPAYTPQ
D. melanogaster    SRAEMDTKRP--PLLAENPST-------------------QYGI---ANASGAPKTYREL

Homo sapiens       AAEGHYTVSYGTDSGEFSSVGEEFYRNHSQPPPLETLGLDADELILIPNGVQIFFVNPAG
D. melanogaster    AA-GLRELLAV---RDAKVLLDELFR----------------------AQVKMYRIEASG

Homo sapiens       EVSAPSYP--------------GYLRIVRFLDNSLDTVLNRPPGFLQVCDWLYPLVPDRS
D. melanogaster    SVTTISGSSTMSLVMCTVGGKWKYLSGIYFIQC-------SMPNEGTAGIWLYPLVPSIT

Homo sapiens       PVLKCTAGAYMFPDTMLQAAGCFVGVVLSSELPEDDRE-LFEDLLRQMSDLRLQANWNRA
D. melanogaster    NCYQTEYGAFIFPDMECQQPGNAFGLMLTKEGQTSRTEDELEDLQQFFLDLLE-A-----

Homo sapiens       EEENEFQIPGRTRPSSDQLKEASGTDVKQLDQGNKDVRHKGKRGKRAKDTSSEEVNLSHI
D. melanogaster    ------VLAG---------------TVVQLKSPT------SQRAGL--------------

Homo sapiens       VPCEPVPEEKPKELPEWSEKVAHNILSGASWVSWGLVKGAEITGKAIQKGASKLRERIQP
D. melanogaster    ----------ASDTVSGSEQVSRHIVSAADFIASNLVRGAEKTGGFMLRSTPYIISKMTP

Homo sapiens       EEKPVEVSPAVTKGLYIAKQATGGAAKVSQFLVDGVCTVANCVGKELAPHVKKHGSKLVP
D. melanogaster    ASMDAQVPSSVQTSVEVAQKVTHAAAGMTGWIAGKVGTASMAVGRYLAPHIQEQGSKLLQ

Homo sapiens       ESLKKDK-DGKSPLDGAMVVAASSVQGFSTVWQGLECAAKCIVNNVSAETVQTVRYKYGY
D. melanogaster    KGFGYDTSEANSTMEGAMTIAAGAVEGVSTVFDGLETSAKILGSSLSENSVKIIEHKYGQ

Homo sapiens       NAGEATHHAVDSAVNVGVTAYNINNIGIKAMVKKTATQTGHTLLEDYQIVDNSQREN--Q
D. melanogaster    QTGNLASGTFDTVGNVFVVSQNVNYITPKGIAKKMVKRTGEAVVSDYKRDLRKSESHYIN

Homo sapiens       EGAANVNVRGEKDEQTKEVKEAKKKDK
D. melanogaster    AGSLYPDLRALKE--------------
```

Domains: MIT UBR PSD

Conservation between groups of similar properties - roughly equivalent to scoring = > 0 in the Gonnet PAM 250 matrix

Positions of fully conserved residue

Supplementary Figure 2. Spartin is highly conserved from fly to human. Alignment of *Drosophila* and human Spartin. Identical residues are indicated with dark gray background. Similar residues are indicated by light gray background. The microtubule-interacting and trafficking (MIT) domain is marked green, ubiquitin binding region (UBR) in blue and plant related senescence domain (PSD) in red.

Supplementary Figure 3

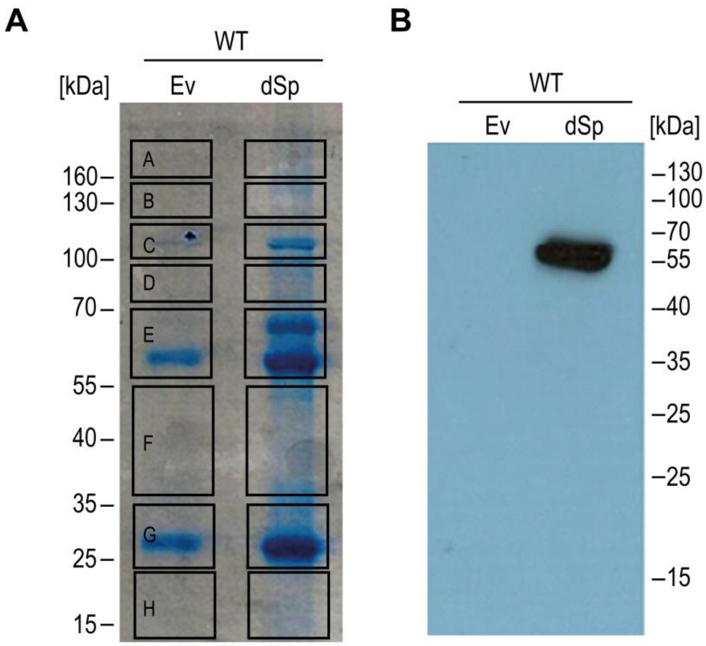

Supplementary Figure 3. FLAG-Pulldown of dSpartin. *A*. Eluate from FLAG (FLAG beads) pull down on SDS gel coomassie stained. Letters correspond to bands on gel in Table *B*. and respective western blot analysis. The blot was probed with FLAG antibody and the corresponding secondary antibodies.

Supplementary Figure 4

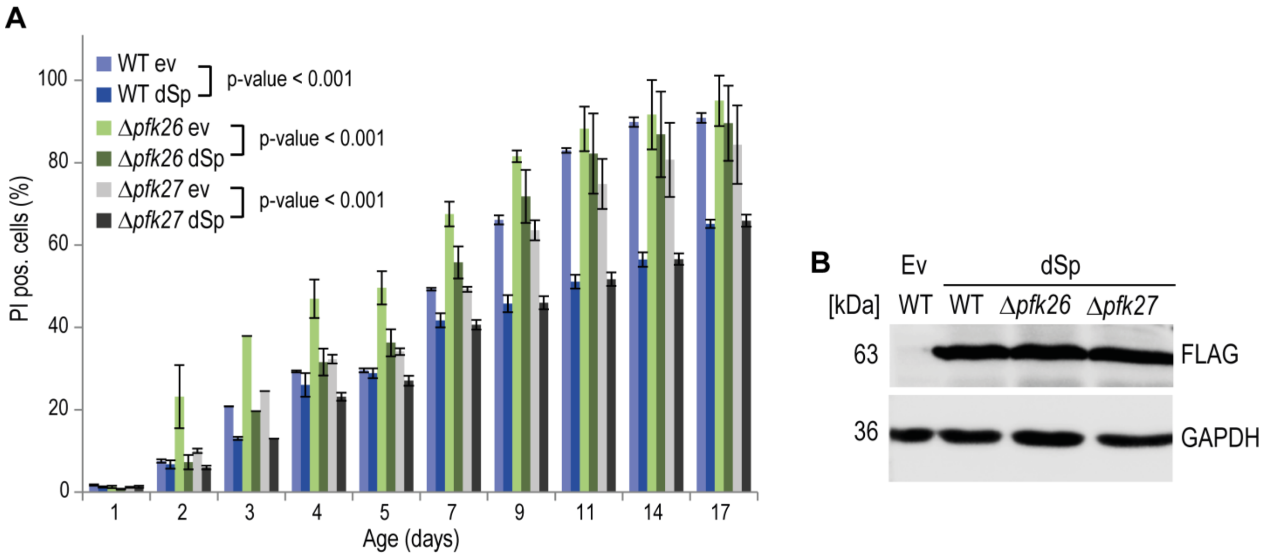

Supplementary Figure 4. *S*partin displays a pro survival role depending on Pfk26 in old cells and complements Δ*pfk26* in young cells but is independent of Pfk27. *A*. Cell death during chronological aging of WT cells expressing dSp or cells harboring the corresponding empty vector control (ev). Dead cells are stained with propidiumiodide (PI). Data represent mean values ± SEM of at least four independent experiments performed at the same time. *B.* Western blot analysis of dSp expression in WT, Δ*pfk26* and Δ*pfk27* cells. The blot was probed with FLAG and as a loading control with glyceraldehyde-3-phosphate dehydrogenase (*GAPDH*) antibody and the corresponding secondary antibodies.

Supplementary Figure 5

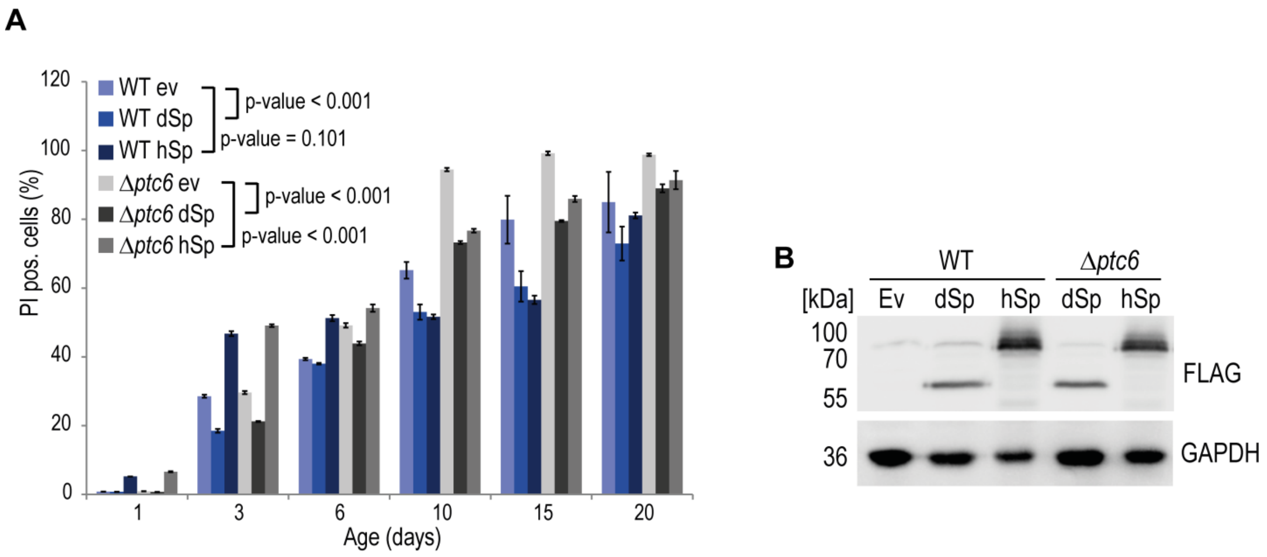

Supplementary Figure 5. *Drosophila* and human spartin promote longevity in yeast and complement the Δ*ptc6* survival phenotype. *A*. Survival during chronological aging of WT cells expressing *Drosophila* (dSp), human spartin (hSp) or cells harboring the corresponding empty vector control (ev). Dead cells are stained with propidiumiodide (PI). Data represent mean values ± SEM of at least four independent experiments performed at the same time. *B.* Western blot analysis of dSp and hSp expression in WT and Δ*ptc6* cells. The blot was probed with FLAG and as a loading control with glyceraldehyde-3-phosphate dehydrogenase (*GAPDH*) antibodies and the corresponding secondary antibodies.

Supplementary Figure 6

A

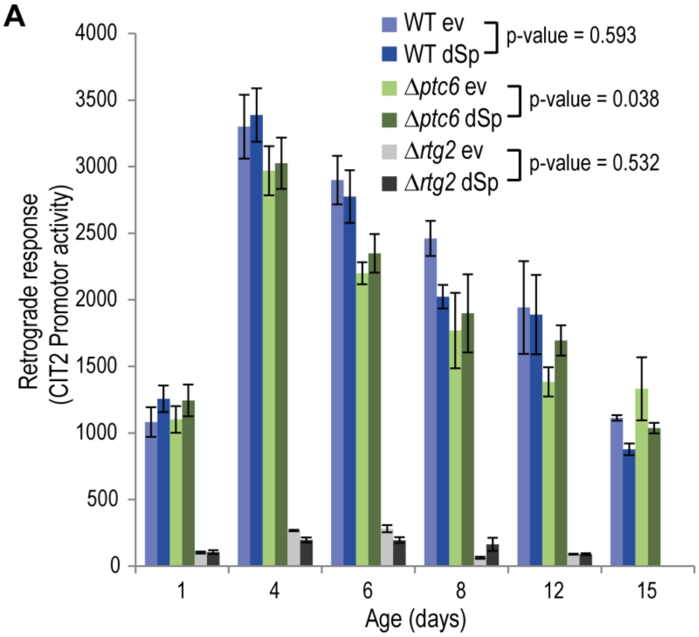

B

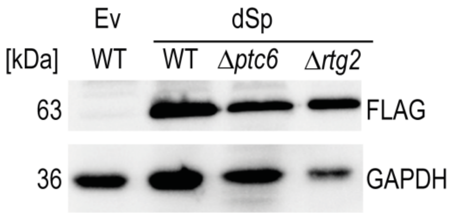

Supplementary Figure 6. Spartin does not influence retrograde response (RTG) signaling in yeast. *A*. Quantification of RTG activity by determining transcriptional activation of an integrated *CIT2-lacZ* reporter gene in WT, Δ*ptc6* and Δ*rtg2* cells expressing dSp or harboring the corresponding ev, additionally carrying pYEp355-*CIT2*::*lacZ*. β-Galactosidase activity was measured from cells at indicated time points during chronological aging. Data represent mean ± SEM of at least three independent experiments performed at the same time. *B.* Western blot analysis of dSp expression in WT, Δ*ptc6* and Δ*rtg2* cells. The western blot was probed with FLAG and as a loading control glyceraldehyde-3-phosphate dehydrogenase (*GAPDH*) antibodies and the corresponding secondary antibodies.
